# Candidate Genes in Testing Strategies for Linkage Analysis and Bioinformatic Sorting of Whole Genome Sequencing Data in Three Small Japanese Families with Idiopathic Superior Oblique Muscle Palsy

**DOI:** 10.3390/ijms23158626

**Published:** 2022-08-03

**Authors:** Toshihiko Matsuo, Chaomulige, Mary Miyaji, Osamu Hosoya, Akira Saito, Kazuyuki Nakazono

**Affiliations:** 1 Graduate School of Interdisciplinary Science and Engineering in Health Systems, Okayama University, Okayama 700-8558, Japan; baochaomulige@s.okayama-u.ac.jp; 2 Department of Ophthalmology, Okayama University Hospital, Okayama 700-8558, Japan; 3 Department of Medical Neurobiology, Graduate School of Medicine, Dentistry and Pharmaceutical Sciences, Okayama University, Okayama 700-8558, Japan; mmiyaji@okayama-u.ac.jp (M.M.); hosoya@okayama-u.ac.jp (O.H.); 4 StaGen Co., Ltd., Tokyo 111-0051, Japan; saito@stagen.co.jp (A.S.); nakazono@stagen.co.jp (K.N.)

**Keywords:** whole genome sequencing, idiopathic superior oblique muscle palsy, strabismus, esotropia, exotropia, linkage analysis, single nucleotide variations and short insertions/deletions, SNVs/InDels, *SSTR5-AS1*, bioinformatics, muscle hypoplasia (aplasia)

## Abstract

Idiopathic superior oblique muscle palsy is a major type of paralytic, non-comitant strabismus and presents vertical and cyclo-torsional deviation of one eye against the other eye, with a large vertical fusion range and abnormal head posture such as head tilt. Genetic background is considered to play a role in its development, as patients with idiopathic superior oblique muscle palsy have varying degrees of muscle hypoplasia and, rarely, the complete absence of the muscle, that is, aplasia. In this study, whole genome sequencing was performed, and single nucleotide variations and short insertions/deletions (SNVs/InDels) were annotated in two patients each in three small families (six patients in total) with idiopathic superior oblique muscle palsy, in addition to three normal individuals in one family. At first, linkage analysis was carried out in the three families and SNVs/InDels in chromosomal loci with negative LOD scores were excluded. Next, SNVs/InDels shared by the six patients, but not by the three normal individuals, were chosen. SNVs/InDels were further narrowed down by choosing low-frequency (<1%) or non-registered SNVs/InDels in four databases for the Japanese population, and then by choosing SNVs/InDels with functional influence, leading to one candidate gene, *SSTR5-AS1* in chromosome 16. The six patients were heterozygous for 13-nucleotide deletion in *SSTR5-AS1*, except for one homozygous patient, while the three normal individuals were wild type. Targeted polymerase chain reaction (PCR) and direct sequencing of PCR products confirmed the 13-nucleotide deletion in *SSTR5-AS1*. In the face of newly-registered *SSTR5-AS1* 13-nucleotide deletion at a higher frequency in a latest released database for the Japanese population, the skipping of low-frequency and non-registration sorting still resulted in only 13 candidate genes including *SSTR5-AS1* as common variants. The skipping of linkage analysis also led to the same set of 13 candidate genes. Different testing strategies that consisted of linkage analysis and simple unintentional bioinformatics could reach candidate genes in three small families with idiopathic superior oblique muscle palsy.

## 1. Introduction

Both eyes in humans or animals such as monkeys and cats are aligned with each other to the same target. Eye movement on each side is controlled by six extraocular muscles: four rectus muscles for horizontal and vertical movement and two oblique muscles for vertical and cyclo-torsional movement. The extraocular muscles are innervated by the oculomotor, trochlear, and abducens nerves coming from the brain stem. The movement of both eyes is coordinated to give rise to binocular vision, which consists of simultaneous perception, binocular fusion, and stereopsis as an integrated neural process of the brain. Paralytic (non-comitant) strabismus is caused by muscle diseases or paralysis of the innervated nerves, and presents misalignment of both eyes only in a certain direction of eye gaze. The most frequent causes for paralytic strabismus are traumatic and ischemic nerve palsies, which are designated as acquired palsies.

In contrast with acquired palsies of cranial nerves for eye movement, congenital palsies of extraocular muscles, and hence congenital palsies of the cranial nerves, are rare [1,2]. Congenital superior oblique muscle palsy is the most common entity of non-comitant or paralytic strabismus [3]. Patients with congenital superior oblique muscle palsy can often maintain binocular vision by compensatory head positioning such as head tilt, as well as by a large vertical fusion range. Based on this tendency, some patients are diagnosed with decompensated superior oblique muscle palsy at a later age in adulthood only when they begin to experience diplopia or eye fatigue. Therefore, idiopathic superior oblique muscle palsy is used as a term to include congenital and decompensated conditions [1].

The hypoplasia of the trochlear nucleus and the nerve, leading to the hypoplasia or aplasia of the superior oblique muscle, has been considered to underlie the development of idiopathic superior oblique muscle palsy [4,5,6,7,8]. An environmental factor such as birth injury might also play a role in the development of idiopathic superior oblique muscle palsy. Congenital fibrosis of the extraocular muscles, also known as congenital cranial disinnervation disorders, belongs to the collection of rare Mendelian disorders, which show varying degrees of restrictive eye movement and eyelid ptosis [2,3]. Idiopathic superior oblique muscle palsy might be a milder type that only affects the trochlear nerve in the entity of congenital cranial disinnervation disorders [5,6,7,8]. Based on this working hypothesis, we previously reported a candidate gene approach in three small families with idiopathic superior oblique muscle palsy [9]. In this study, whole genome sequencing was done in nine individuals of these three small families with idiopathic superior oblique muscle palsy, and single nucleotide variations and short insertions/deletions (SNVs/InDels) were annotated and narrowed down to reach a candidate gene by a testing strategy that combined linkage analysis and a database-dependent bioinformatic approach. Different testing strategies were also challenged in this study to reach candidate genes by way of skipping the linkage analysis or skipping low-frequency or non-registration sorting of SNVs/InDels.

## 2. Results

### 2.1. Phenotype in Three Families

**Figure 1:**
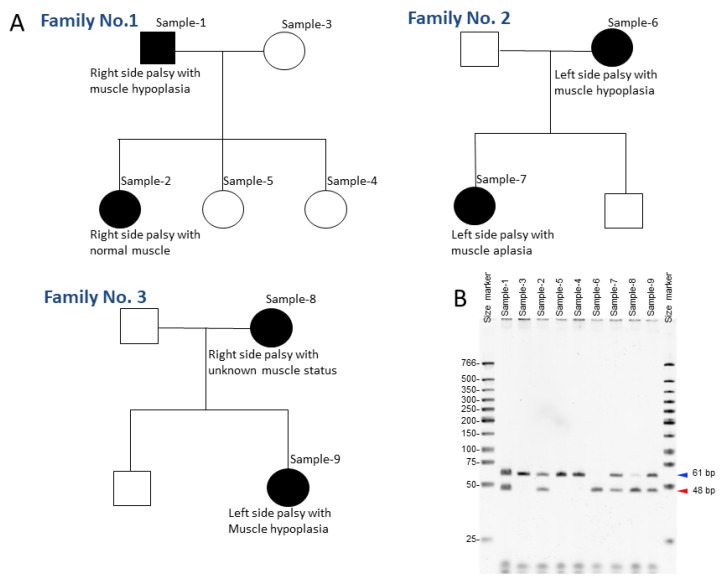
(**A**). Pedigrees for three families with idiopathic superior oblique muscle palsy. (**B**). Polyacrylamide gel electrophoresis of targeted polymerase chain reaction of genomic DNA for *SSTR5-AS1* 13-necleotide deletion in nine individuals of the three families.

**Figure 2:**
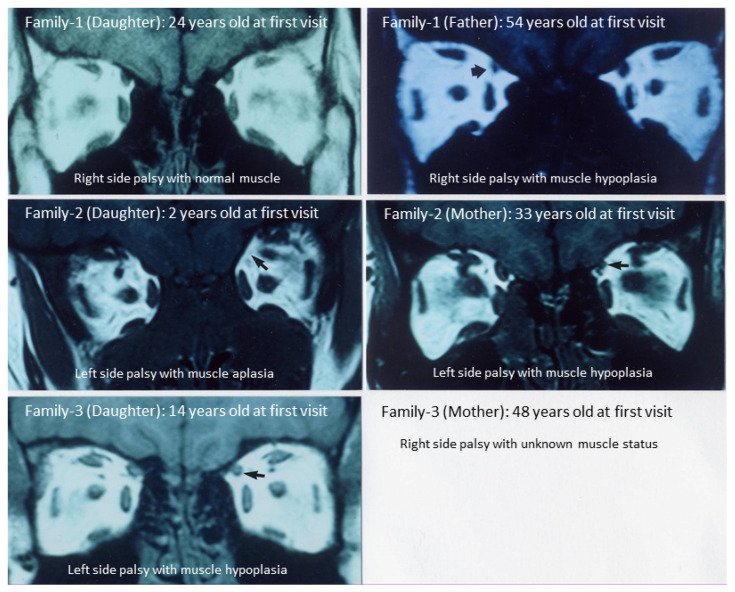
Coronal sections of magnetic resonance imaging in patients with idiopathic superior oblique muscle palsy in three families. One patient (Family 2, Daughter) shows superior oblique muscle aplasia (arrow), while three patients show hypoplasia of the muscle (arrows). Modified from reference [9].

Participants in this study were nine individuals including six patients with idiopathic superior oblique muscle palsy and three normal individuals in three families seen at Okayama University Hospital (Figure 1A, Table 1). As reported previously [9], three patients showed superior oblique muscle hypoplasia, while one patient showed muscle aplasia (Figure 2). Genomic DNA was available for a father and a daughter, both of which were diagnosed as idiopathic superior oblique muscle palsy, as well as of a mother and two daughters with the normal phenotype in Family 1. Genomic DNA was obtained from a mother and a daughter with idiopathic superior oblique muscle palsy in each of Family 2 and Family 3 (Figure 1A).

**Table 1:** Clinical characteristics of six patients in three families with idiopathic superior oblique muscle (SO) palsy.

Family	Member	Laterality of SO Palsy	Abnormal Head Posture	Deviation at 5 m (Δ)	Bagolini Striated Glasses Test		TNO Test	Surgical Procedure
					at 5 m	at 0.3 m		
1	Father	Right	Head tilt to Left	30ΔRHT	Fusion	Diplopia	Absent	Left IR recession
			10 degrees	6ΔX				4 mm
	Daughter	Right	Head tilt to Left	25ΔRHT	Fusion	Fusion	60 s	Right IO recession
			5–10 degrees	10ΔX				
2	Mother	Left	Head tilt to Right	20ΔLHT	Left eye	Left eye	Absent	No surgery
			10 degrees	6ΔX	suppression	suppression		
	Daughter	Left	Head tilt to Right	25ΔLHT	* unknown	* unknown	Absent	Left IO recession
			20 degrees	12ΔET				
3	Mother	Right	Head tilt to Left	14ΔRHT	Right eye	Fusion	60 s	Right IO recession
			5–10 degrees	10ΔX	suppression			
	Daughter	Left	Head tilt to Right	12ΔLHT	Right eye	Fusion	240 s	No surgery
			5 degrees	16ΔXT	suppression			

Δ, prism diopter; s, second of arc; RHT, right hypertropia; LHT, left hypertropia; X, exophoria; XT, exotropia; ET, esotropia; IR, inferior rectus muscle; IO, inferior oblique muscle. * unknown because of the young age at 2 years. Modified from [9].

### 2.2. Parametric Linkage Analysis with SNVs

SNVs data with normal call at 7,941,582 loci obtained by the whole genome sequencing in nine individuals of three families (Figure 1A) were used for linkage analysis. The quality of SNVs in each individual was assessed by PLINK software and determined as a call rate equal to and greater than 95%, leading to the conclusion that no individual was excluded. Next, identity-by-decent (IBD) values between paired individuals were calculated to prove no overlapping of the same individual. Finally, 677,045 loci of SNVs with a call rate of less than 0.8 and 770,122 loci of SNVs with monotype with minor allele frequency = 0 were excluded. SNVs with genetic incongruence, based on family information, were checked by the software PEDSTATS, and 128,683 loci were excluded as suspicious of poor quality.

In linkage analysis, 12,835 loci of SNVs were chosen at the interval of about 0.5 cM (centimorgan) between neighboring loci, based on genetic maps for the 1000 Genome Project variants using the tool “hotspots”. The allele frequency in SNVs selected as above was calculated by Japanese Genotype in the 1000 Genome Project. Three modes of inheritance were set as dominant (a/a = 0.99, a/A = 0.99, A/A = 0.01), additive (a/a = 0.99, a/A = 0.5, A/A = 0.01), and recessive (a/a = 0.99, a/A = 0.01, A/A = 0.01), based on the minor allele frequency set at 0.0001 (“a” as a variant allele and “A” as a wild-type allele). No loci showed a logarithm of the odds (LOD) score with significance, equal to or greater than 3.3.

### 2.3. SNVs/InDels

**Figure 3:**
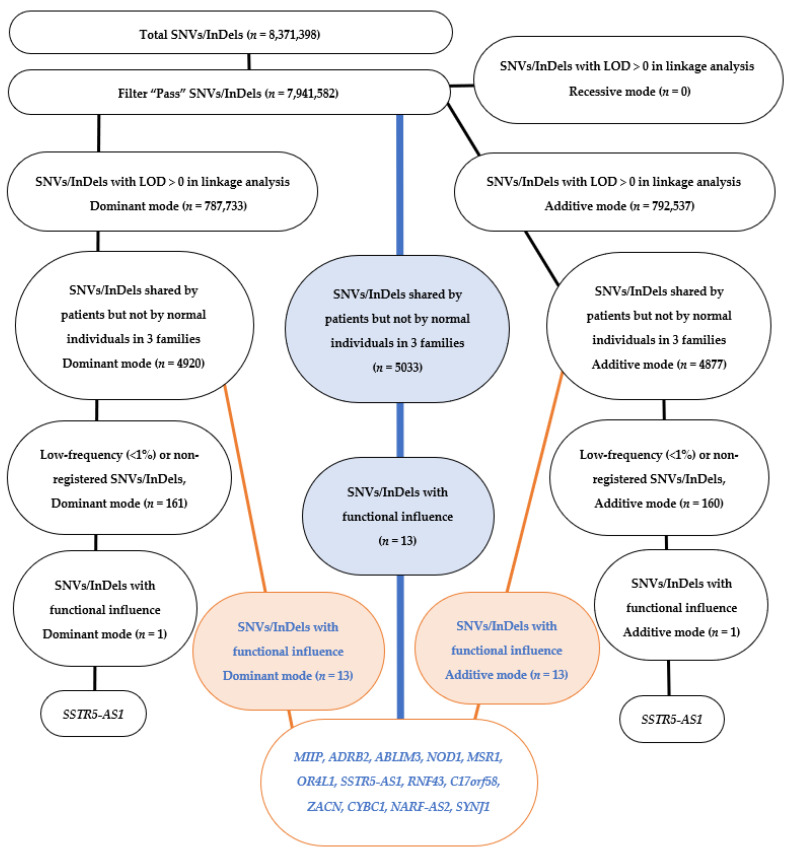
Flow chart for narrowing-down of single nucleotide variations and short insertions and deletions (SNVs/InDels), which are detected by whole genome sequencing in nine members of three families with idiopathic superior oblique muscle palsy. Orange lines and orange boxes indicate skipping of low-frequency (<1%) or non-registration sorting. A blue thick line and blue boxes indicate skipping of narrowing-down by linkage analysis. Note that the skipping of linkage analysis reaches the same set of 13 candidate genes as the skipping of low-frequency (<1%) or non-registration sorting.

The number of SNVs/InDels in the variant call format (VCF) file was 8,371,398, and 7,941,582 SNVs/InDels were chosen by the process of Filter “Pass” (Figure 3). In these 7,941,582 SNVs/InDels, loci with zero and negative values of LOD scores in linkage analysis were used first to exclude the non-relevant SNVs/InDels. The number of SNVs/InDels located at loci with positive values of LOD scores in the linkage analysis was 787,733 in the dominant mode, 792,537 in the additive mode, and zero in the recessive mode of inheritance. Candidate SNVs/InDels that were shared by all six patients and further not shared by all three normal individuals were 4920 of 787,733 in the dominant mode and 4877 of 792,537 in the additive mode (Figure 3).

From these candidate SNVs/InDels, low-frequency alleles were chosen based on the criteria that allele frequency was less than 1% or that the variant in an allele has not be registered in the databases for Japanese: 1000 Genomes JPT (*n* = 104), Human Genetic Variation Database (HGVD, *n* = 1208), HapMap JPT (*n* = 86), and Integrative Japanese Genome Variation Database (iJGVD, *n* = 1070), resulting in 161 SNVs/InDels in the dominant mode and 160 in the additive mode. These low-frequency SNVs/InDels were narrowed down by the expectation of their functional influence: frameshift, nonsense, readthrough, insertion-deletion, deletion, insertion, missense, splice donor site, or splice acceptor site in the function in the annotation information (Figure 3).

Only one SNV/InDel left behind in the process of the narrowing-down was *SSTR5-AS1*, which was common in the dominant mode and the additive mode (Figure 3). The splice acceptor site in the *SSTR-AS1* had 13-nucleotide deletion from the chromosomal positions starting at 1,117,031 and ending at 1,117,043, including CTTTCCATATAGC. All six patients except for one with the homozygote (mother in Family 2) were heterozygous for this 13-nucleotide deletion (Table 1). Of course, the three normal individuals in Family 1 had no deletion on both chains. LOD scores at the locus harboring the 13-nucleotide deletion of *SSTR5-AS1* were 0.1989 in dominant mode and 0.0387 in additive mode.

### 2.4. CNVs and SVs

CNVnator detected 17,873 copy number variations (CNVs), while CNVkit detected 321 CNVs. Candidate CNVs shared by all six patients, but not by three normal individuals in Family 1, were narrowed down to seven CNVs in CNVnator and none in CNVkit, respectively. For structural variations (SVs), four candidate SVs with a breaking point in a specific gene were narrowed down in the same manner from 17,755 SVs detected by Manta, and 174 candidate SVs were narrowed down from 2,174,705 SVs detected by GRIDSS.

### 2.5. Targeted Polymerase Chain Reaction (PCR) and Direct Sequencing

**Figure 4:**
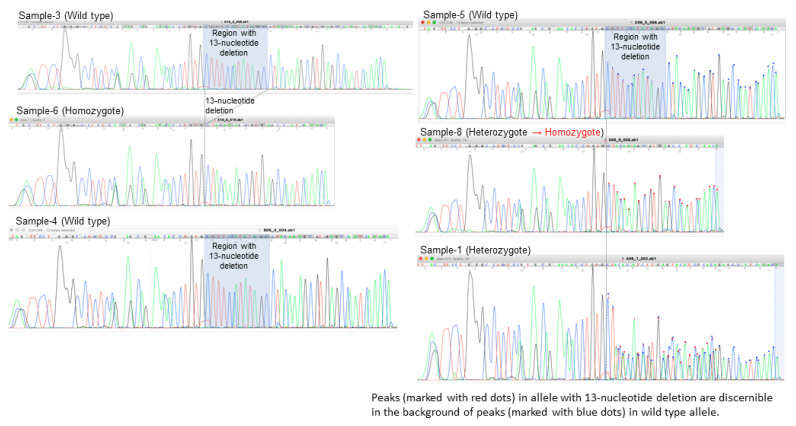
Direct sequencing of polymerase chain reaction (PCR) products for *SSTR5-AS1* 13-necleotide deletion in members of three families with idiopathic superior oblique muscle palsy.

To confirm the results of whole genome sequencing, the 13-nuecleotide deletion in *SSTR5-AS1* was tested by targeted PCR (Figure 1B). The heterozygosity for the 13-nucleotide deletion in five patients was consistent between whole genome sequencing and targeted PCR (Figure 1B), while one patient (Sample 8, Mother in Family 3) heterozygous for the 13-nucleotide deletion in whole genome sequencing was disclosed as homozygous for the deletion by direct sequencing of the PCR product (Figure 4, Table 2). The other patient (Sample 6, Mother in Family 2) homozygous for the 13-nucleotide deletion was confirmed as such by targeted PCR and direct sequencing (Figure 4).

To search further for the role of the 13-nucleotide deletion in *SSTR5-AS1*, targeted PCR was performed in 104 unrelated patients with idiopathic superior oblique muscle palsy as well as 233 unrelated patients with esotropia (*n* = 117) or exotropia (*n* = 116), which served as the control (Table 3). The rate of the homozygote was significantly higher and the rate of the heterozygote regarding the 13-nucleotide deletion in *SSTR5-AS1* in the patients with idiopathic superior oblique muscle was significantly lower compared with patients with esotropia or exotropia (*p* = 0.0022, chi-square test, Table 3).

### 2.6. Testing Strategy for Different Ways of Sorting

In the first of the various sorting methods, the step of low-frequency (<1%) or non-registration sorting was skipped, and SNVs/InDels was narrowed down only by the step of functional influence sorting. In this flow of sorting (Figure 3), the same set of 18 SNVs/InDels, corresponding to 13 candidate genes (*MIIP*, *ADRB2*, *ABLIM3*, *NOD1*, *MSR1*, *OR4L1*, *SSTR5-AS1*, *RNF43, C17orf58*, *ZACN*, *CYBC1*, *NARF-AS2,* and *SYNJ1*), was obtained in both the dominant mode and the additive mode. Table S1 shows the allele frequency of these 18 SNVs/InDels in different databases for the Japanese population, which comprises the latest released jMorp, in addition to the originally used databases of 1000 Genomes JPT, HGVD, HapMap JPT, and iJGVD. The allele frequency of the 18 SNVs/InDels ranges from 0.2 to 0.5 as common variants. Table S1 also summarizes the functional predictions of the SNVs using various methods. Table 4 shows the reported function and disease association of the 13 candidate genes, corresponding to the 18 SNVs/InDels.

In the second sorting method, the sorting of SNVs/InDels with LOD > 0 in linkage analysis was skipped to reach the same final set of 18 SNVs/InDels (13 candidate genes) as shown above in the first sorting method. In the process of narrowing-down, 5033 SNVs/InDels were obtained by the sorting of SNVs/InDels, which were shared by patients, but not by normal individuals in three families, and then 18 SNVs/InDels (13 candidate genes) were obtained by functional influence sorting alone (Figure 3).

In the third sorting method, we changed the condition of narrowing-down at the step to obtain SNVs/InDels shared only by the six patients, irrespective of the three normal individuals. In the dominant mode, the number of SNVs/InDels was 195,762 at the step of sharing by the six patients, 20,761 at the step of low-frequency (<1%) or non-registration sorting, and 25 at the step of functional influence sorting. In the additive mode, the number of SNVs/InDels was 196,928 at the step of sharing by the six patients, 20,870 at the step of low-frequency (<1%) or non-registration sorting, and 25 at the step of functional influence sorting. In both modes of inheritance, the narrowing-down, based on the new condition, led to the same set of 22 genes (3 of 25 SNVs/InDels are located on the same gene): *PRAMEF25/PRAMEF26*, *PRAMEF14*, *AL355149.2*, *NBPF3*, *CDC42*, *PPP2R2B*, *MMD2*, *TNRC18*, *UMAD1, RP1L1*, *FDFT1*, *GOLGA8I*, *GOLGA6L2*, *GOLGA8R*, *LOC100996413*, *SSTR5-AS1*, *TPSB2*, *LOC105371045*, *ARHGAP27P1-BPTFP1-KPNA2P3/hsa-mir-6080*, *FADS6*, *SIRPA*, and *FKSG68.*

## 3. Discussion

This study was designed to test a strategy for combining linkage analysis with bioinformatic sorting to narrow down SNVs/InDels detected by whole genome sequencing in three small families with idiopathic superior oblique muscle palsy. In our initial testing strategy, SNVs/InDels located in the chromosomal loci with negative values of LOD scores in linkage analysis were excluded at the first step. Owing to the limited number of affected or unaffected individuals in small families, linkage analysis could not reach a locus with a significant LOD score. On the assumption that loci with negative LOD scores would not harbor responsible genes for the phenotype, we chose SNVs/InDels that were located in loci with positive LOD scores. We then made the second assumption that the phenotype of the three small families would result from the common genotype as these families came from the same local area in the western part of the mainland Honshu of Japanese archipelago. Based on this second assumption, we chose SNVs/InDels shared by six affected individuals in the three families and further not shared by the three unaffected individuals in Family 1. The narrowing-down of SNVs/InDels in the following process was carried out by standard procedures: the choice of low-frequency (<1%) or non-registered SNVs/InDels in the databases of Japanese population and then the choice of SNVs/InDels with functional influence (Figure 3).

To our surprise, the simple unintentional bioinformatics, as mentioned above in the present study, reached a single InDel, 13-nucleotide deletion of *SSTR5-AS1*, heterozygous in all patients except for one who was homozygous (Sample 6, Family 2, Mother). As the next step, we designed forward and reverse primers for targeted polymerase chain reaction to sandwich this 13-nucleotide deletion of *SSTR5-AS1*. Polyacrylamide gel electrophoresis of PCR products confirmed the wild type, heterozygosity, and homozygosity of the 13-nucleotide deletion (Figure 1B). In direct sequencing of PCR products, one patient (Sample 8, Family 3, Mother) who was heterozygous for the 13-nucleotide deletion with a faint band of the non-deletion counterpart was revealed as homozygous for the 13-nucleotide deletion. In summary, two patients in Family 1 were heterozygous, while three unaffected individuals were wild type regarding the 13-nucleotide deletion of *SSTR5-AS1*. In Family 2 and Family 3, affected mothers were homozygous, while affected daughters were heterozygous for the 13-nucleotid deletion of *SSTR5-AS1* (Table 2). It should, however, be reserved that the mother in Family 3 could be heterozygous as the number of reads for the deletion was 15 and the number of reads for the non-deletion wild type was 17 in whole genome sequencing of Sample 8.

To search for the role of the 13-nucleotide deletion of *SSTR5-AS1*, genomic DNA of 104 unrelated patients with idiopathic superior oblique muscle palsy was typed by targeted polymerase chain reaction. Roughly, one-quarter was the wild type, one-quarter was the homozygote, and two-quarters were the heterozygote (Table 3). As a kind of control, genomic DNA of 233 patients with esotropia or exotropia was typed to demonstrate that the rates of the wild type, homozygote, and heterozygote were significantly different from the rates in patients with idiopathic superior oblique muscle palsy. The rates of the genotypes were almost the same between the patients with esotropia and exotropia. Ideally, the genotypes of an eye disease should be compared with the genotypes of the normal population or at least of patients with a non-eye disease. In the background of choosing low-frequency (<1%) or non-registered SNVs/InDels in the narrowing-down process, the allele frequency of the 13-nucleotide deletion of *SSTR5-AS1*, estimated from the heterozygote and homozygote, was high in both patients with idiopathic superior oblique muscle palsy and patients with esotropia or exotropia. These results suggest that idiopathic superior oblique muscle palsy and comitant strabismus including esotropia and exotropia might share a genetic background to some extent, which remains to be determined.

In contrast with idiopathic superior oblique muscle palsy as paralytic (non-comitant) strabismus, comitant strabismus presents the same degree of deviation in all directions of gaze as the misalignment of both eyes. This diagnostic hallmark is used to differentiate comitant strabismus from non-comitant or paralytic strabismus. Esotropia and exotropia are predominant entities of comitant strabismus that show inward and outward deviation of the eyes, respectively, in the horizontal direction [10,11]. Comitant strabismus has an onset in the earlier years of life and is a multifactorial disease with a genetic and environmental background [12,13,14]. Twin studies [15] and family pedigree analyses [16,17,18,19,20] have supported the role of genetic background in comitant strabismus, while hypoxia during pregnancy and delivery might be an environmental risk factor for the development of comitant strabismus [13,14]. Genome-wide association studies for comitant strabismus were also conducted [21,22,23]. Until now, idiopathic superior oblique muscle palsy has been recognized as an independent disease from comitant strabismus. The present results suggest that common genetic background to an unknown extent might be shared by idiopathic superior oblique muscle palsy and comitant strabismus. Caution must be taken against this suggestion as no other evidence until now has supported the common genetic background for comitant strabismus and idiopathic superior oblique muscle palsy.

In this study, we used four databases for the Japanese population: 1000 Genomes JPT, HGVD, HapMap JPT, and iJGVD, and we confirmed that the 13-nucleotide deletion of *SSTR5-AS1* gene was not registered in either database. To face the rather high rates of 13-nucleotide deletion of the *SSTR5-AS1* gene by targeted polymerase chain reaction in unrelated patients with idiopathic superior oblique muscle palsy as well as unrelated patients with esotropia or exotropia (Table 3), we searched for a newly released database, jMorp (Japanese Multi Omics Reference Panel, 14K JPN, *n* = 14,129), for the Japanese population, which is the expanded version of iJGVD in Tohoku Medical Megabank Organization (ToMMo), and found that the 13-nucleotide deletion of *SSTR5-AS1* gene has been newly registered at the allele frequency of 0.51306. The genotypes of the 13-nucleotide deletion of *SSTR5-AS1* in jMorp are distributed as 3360 (23.8%) in the wild type, 7040 (49.8%) in the heterozygote, and 3729 (26.4%) in the homozygote. The genotypes of 104 patients with idiopathic superior oblique muscle palsy (Table 3) are basically similar to the genotypes of people in jMorp. Tohoku Medical Megabank has been collecting habitants in Tohoku district, northern part of the mainland, Honshu, in the Japanese archipelago, while the other databases mainly include people in the Tokyo metropolitan area and western part of mainland Honshu. Habitants in the northern part and western part of mainland Honshu are known to have somewhat different genetic backgrounds historically even in the Japanese population. Anyway, the narrowing-down by bioinformatics would cause an intrinsic problem in terms of database which is used for the reference.

According to the newly released database (jMorp), the 13-nucleotide deletion of *SSTR5-AS1* gene has no more fulfilled the criteria for low-frequency (<1%) SNVs/InDels in the process of narrowing-down, and consequently, no gene has been left behind in the process of narrowing-down of SNVs/InDels in the present flow. In a testing strategy for different sorting, we skipped the step of low-frequency (<1%) or non-registration sorting, and narrowed down SNVs/InDels only by the step of functional influence sorting. To our surprise, the new sorting of SNVs/InDels has led to only 13 genes, including *SSTR5-AS1*, that are common in both modes of inheritance (Figure 3, Table 4 and Table S1). The sorting of SNVs/InDels, based on the low-frequency (<1%) or non-registration in the database, is intended to detect rare variants, and might have a risk for missing common variants for the phenotype. In this sense, *SSTR5-AS1*, as a common variant, would remain as a tentative candidate for the phenotype of idiopathic superior oblique muscle palsy.

It must be emphasized again as a limitation in the present linkage study that linkage analysis could not reach a locus with a significant LOD score. The non-significant result would be attributed to the fact that the number of individuals was too small for linkage analysis to arrive at reliable loci with LOD scores. In this study, we used linkage analysis to obtain hints for selecting tentative loci for candidate genes. In this aspect of linkage study, the whole genome sequencing was performed only in the members of the three families with idiopathic superior oblique muscle palsy, but not in a cohort of 104 unrelated patients with the disease. The cohort of unrelated patients with idiopathic superior oblique muscle palsy as well as the cohort of unrelated patients with esotropia or exotropia were used for single nucleotide polymorphism (SNP) typing by arrays to conduct a genome-wide association study in another project, which is now in progress.

Accordingly, in the other testing strategy for different sorting, we skipped the sorting by linkage analysis. In the process of narrowing-down, 5033 SNVs/InDels were obtained by the sorting of SNVs/InDels shared by six patients but not by three normal individuals in three families, and then the same set of 18 SNVs/InDels (13 candidate genes) as reached in the initial strategy of linkage analysis at the first step was obtained by functional influence sorting alone (Figure 3, Table 4 and Table S1). This fact suggests that linkage analysis would not contribute to the narrowing down of SNVs/InDels and that the supposed common segregation of the genotype–phenotype in the small families would be a key in the sorting to reach candidate genes. Furthermore, it should be emphasized that the functional sorting would play a key role in narrowing down the SNVs/InDels in this study.

The clear segregation of the 13-nucleotide deletion of *SSTR5-AS1* gene with the phenotype in the three families with idiopathic superior oblique muscle palsy is naturally based on the selection of these SNVs/InDels shared by six patients in the three families, and not shared by three normal individuals in Family 1. As a common understanding in linkage analysis, we assume in the present study that six patients in three families with idiopathic superior oblique muscle would share common SNVs/InDels, which might be related to muscle hypoplasia or aplasia. As the other limitation of this study, it might be inappropriate to assume that SNVs/InDels shared by six patients and not shared by three normal individuals would be candidates for the phenotype of idiopathic superior oblique muscle palsy in the three families. In the case that this phenotype would show incomplete penetrance, the narrowing-down process of SNVs/InDels at this step would lead in the wrong direction. It should be stated as a general limitation that the normal population or the normal phenotype is indeed difficult to define from the viewpoint of the disease status such as idiopathic superior oblique muscle palsy. Idiopathic superior oblique muscle palsy has, in fact, a wide range of spectrum of the phenotype, which would have continuous transition from the so-called normal condition [5]. Based on this way of thinking, we changed the condition of narrowing down at the step to show SNVs/InDels shared only by the six patients, irrespective of the three normal individuals, in a different testing strategy. In the process of low-frequency (<1%) or non-registration sorting, and then functional influence sorting, the same 22 genes were obtained in the dominant mode and additive mode of inheritance.

Even though methodological limitations are present in this study, *SSTR5-AS1* gene might be considered as one candidate among the 13 candidate genes as common variants for the phenotype of idiopathic superior oblique muscle palsy. *SSTR5-AS1* gene on chromosome 16 (16p13.3) is a long non-coding RNA and would play a role in regulating the expression of other genes [24]. Until now, the role of *SSTR5-AS1* has been studied in diabetes [24] as well as in different kinds of cancers [25,26,27,28,29,30]. As the literature is scarce on the role of *SSTR5-AS1*, the present result could not be supported by any functional argument. It should also be noted that there are no data of expression analysis in different genotypes of *SSTR5-AS1*, regarding the effect of its 13-nucleotide deletion, in jMorp. It appears that no potential physiological relationship would be present between the function of the 13 candidate genes and the phenotype of idiopathic superior oblique muscle palsy (Table 4). Under the circumstances, these 13 genes are known to be expressed in the brain, suggestive of their roles in the phenotype. In the present study, the whole genome sequencing confirmed *ARIX* and *PHOX2B* polymorphisms in the three families reported in our previous study as a candidate gene approach [9]. The phenotypic difference in idiopathic superior oblique muscle palsy would be correlated with the genotypic difference of *SSTR5-AS1* in the cohort of unrelated patients with the palsy [8]. Future studies will be required to understand the supposed role of *SSTR5-AS1* in the development of idiopathic superior oblique muscle palsy.

## 4. Materials and Methods

### 4.1. The Participants

The participants in this study were nine individuals including six patients with idiopathic superior oblique muscle palsy and three normal individuals in three families seen at Okayama University Hospital (Table 1, Figure 1A) [9]. In addition to the nine members in the three families, polymerase chain reaction targeted to a specific gene was carried out in 104 unrelated patients with idiopathic superior oblique palsy, 117 patients with esotropia, and 116 patients with exotropia. Genomic DNA was isolated from leukocytes of 10 mL peripheral blood in each patient following written consent to be involved in the study [6,7,8,9]. The study conformed to the tenets of the Declaration of Helsinki and was approved by the Ethics Committee of Okayama University Graduate School of Medicine, Dentistry, and Pharmaceutical Sciences and Okayama University Hospital (Identifier 1512-027).

### 4.2. Whole Genome Sequencing

Genomic DNA in each individual passed the quality check by concentration measurement with Qubit dsDNA BR Assay Kit by Qubit 3.0 Fluorometer (Thermo Fisher Scientific, Waltham, MA, USA) and 1% agarose gel electrophoresis, which showed a main band around 23 kb with no degradation. Genomic DNA was sheared into approximately 350 bp fragments, and used to build a library with TruSeq Nano DNA Library Prep Kit (Illumina, San Diego, CA, USA). Sequencing was performed on a NovaSeq 6000 platform (Illumina, San Diego, CA, USA) in paired-end 150 bp configuration at Riken Genesis, Co., Ltd. (Tokyo, Japan).

In bioinformatics, the read data were submitted to quality check and cleaning, and mapped by Dragen (version 3.7.5., Illumina) to the reference sequences (UCSC Genome Browser hg19). The following other databases were used: dbSNP (NCBI Build 151), COSMIC (version 88), ClinVar (Release 2019-02), ICGC (Release 27), CCDS (Release 15), RefSeq (UCSC Genome Browser, dumped 20181125), GENCODE (UCSC Genome Browser ENCODE/GENCODE version 19), 1000 Genomes (phase 3 release v5), NHLBI ESP (ESP6500SI-V2), HGVD (version 2.30), HapMap (Release 27), iJGVD (NBDC Research IO hum0015.v1), ExAC (Release 1), DGV (Release 20160515), and ChiTaRS 3.1 (Release 20160816).

Cleaning of the read data was performed by Cutadapt and the original program at Riken Genesis to eliminate (1) the adaptor sequence contaminated at the 3′ end, (2) nucleotide(s) at the 3′ end with a quality value less than 3, and (3) the 6 bp adaptor sequence frequently present at the 3′ end. Reads with a length of less than 20 bp were excluded and reads with low quality were also excluded, based on the criteria of reads: (1) the rate of nucleotides with quality values less than 5 was greater than 10% of the total nucleotides, (2) the rate of N was greater than 10% of the total nucleotides, and (3) the number of the same nucleotide was equal to or greater than the total number of nucleotides minus 3. Finally, reads with pair ends were chosen.

The mapping results were combined and submitted to single nucleotide variations and short insertions and deletions (SNVs/InDels) analysis, copy number variation (CNV) analysis, and structural variation (SV) analysis.

### 4.3. Software in Bioinformatics

The quality of SNP data was evaluated by PLINK [31], pedigree incongruency was checked by PEDSTATS [32], and then linkage analysis was carried out with MERLIN [32]. In the detection of SNVs/InDels, additional annotation was performed by SpliceAI to predict the influence on splicing [33]. CNVs were detected independently by CNVnator [34] and CNVkit [35]. The detected variations with Filter “Pass” were integrated by SURVIVOR [36] and further annotated by AnnotSV [37]. SVs were detected independently by Manta [38] and GRIDSS [39], and the detected variations were annotated by AnnotSV [37]. Functional predictions for SNVs were accomplished by tools in dbNSFP version 4.1 (database for nonsynonymous SNPs’ functional predictions [40,41], as shown in Table S1.

### 4.4. Polymerase Chain Reaction and Direct Sequencing

Polymerase chain reaction (PCR) was performed with 10 ng each genomic DNA by forward primer (F1: TCTCCAGAAGCAGAAACACCTGT for polyacrylamide gel electrophoresis, F2: TTCGCCTTCCACCACGGTTG for agarose gel electrophoresis) and reverse primer (R1: gtttgattggctcacgctttgt), using Q5 Hot Start High-Fidelity DNA Polymerase (New England Biolabs Japan, Tokyo), in 30 cycles of denaturation at 98 °C for 5 s, annealing at 67 °C for 10 s, and elongation at 72 °C for 5 s. PCR products were loaded onto 10% polyacrylamide gel electrophoresis in 1× TBE (89 mM Tris-borate, 2 mM EDTA) buffer and stained with SYBR Green I (TaKaRa, Kusatsu, Japan).

For direct sequencing, PCR products were ethanol-precipitated, resuspended in T10E0.1 buffer (10 mM Tris-borate, 0.1 mM EDTA), and loaded onto 3% agarose gel electrophoresis in 1× TBE buffer. The gel was stained with ethidium bromide, and gel pieces with bands were cut out. DNA was extracted by Qiagen gel extraction kit (Qiagen, Venlo, The Netherlands) and the concentration of DNA was checked by Quant-iT PicoGreen dsDNA Assay kit (Thermo Fisher Scientific, Waltham, MA, USA). Sequencing was performed with BigDye Terminator ver1.1 in Applied Biosystems 3130xl Genetic Analyzer (Thermo Fisher Scientific, Waltham, MA, USA).

## 5. Conclusions

Different testing strategies for linkage analysis in combination with the flow of simple unintentional bioinformatics were used to narrow down SNVs/InDels detected by whole genome sequencing in three small families with idiopathic superior oblique muscle palsy. The common genotype–phenotype supposition in small families and functional influence sorting of SNVs/InDels were two key steps to reach 13 candidate genes, including *SSTR5-AS1*, as common variants. A limitation must be placed at the interpretation of the present results as the narrowing down of SNVs/InDels by bioinformatics would be intrinsically influenced by what kind of database is being referred to. In this sense, the present results obtained by the flow of methods should be recognized as preliminary and would be used as a standpoint for future genetic studies of idiopathic superior oblique muscle palsy.

## Figures and Tables

**Table 2:** *SSTR5-AS1* 13-nucleotide (CTTTCCATATAGC) deletion in three families with idiopathic superior oblique muscle palsy.

Sample Name	Phenotype	Whole Genome Sequencing	Polymerase Chain Reaction (PCR)	Direct Sequencing of PCR Products
Sample 1	Family 1, Father, affected	Heterozygote	Heterozygote	Heterozygote
Sample 2	Family 1, Daughter, affected	Heterozygote	Heterozygote	Unreadable
Sample 3	Family 1, Mother, normal	Wild type	Wild type	Wild type
Sample 4	Family 1, Daughter, normal	Wild type	Wild type	Wild type
Sample 5	Family 1, Daughter, normal	Wild type	Wild type	Wild type
Sample 6	Family 2, Mother, affected	Homozygote	Homozygote	Homozygote
Sample 7	Family 2, Daughter, affected	Heterozygote	Heterozygote	Unreadable
Sample 8	Family 3, Mother, affected	Heterozygote	Hetero/Homozygote *	Homozygote
Sample 9	Family 3, Daughter, affected	Heterozygote	Heterozygote	Unreadable

* Wild type band is faint.

**Table 3:** Genotypes of *SSTR5-AS1* 13-nucleotide deletion, determined by targeted polymerase chain reaction, in unrelated patients with idiopathic superior oblique muscle palsy (SO), esotropia (ET), or exotropia (XT).

Genotype	Phenotype				
*SSTR5-AS1* 13-Nucleotide Deletion	Idiopathic Superior Oblique Muscle Palsy (SO) (*n* = 104)	Esotropia (ET) (*n* = 117)	Exotropia (XT) (*n* = 116)	Esotropia or Exotropia (ET+XT) (*n* = 233)	Chi-Square *p*-Value SO versus ET + XT
Wild type	28 (26.9%)	23	18	41 (17.6%)	
Heterozygote	54 (8 *) (51.9%)	82 (27 *)	84 (22 *)	166 (49 *) (71.2%)	
Homozygote	22 (21.2%)	12	14	26 (11.2%)	0.0022 (0.1462 **)
**Allele frequency**					
Wild type/deletion	110/98	128/106	120/112	248/218	1

(numerals *) in heterozygote indicate that the non-deletion wild-type band is fainter than the deletion band. ** *p*-value when faint non-deletion wild-type bands were considered as artifact and numerals * were included in homozygote.

**Table 4:** Thirteen candidate genes obtained by linkage analysis and bioinformatic sorting of whole genome sequencing data in three families with idiopathic superior oblique muscle palsy.

Genes	Location	Full Name	Function	Diseases in Association
*MIIP*	1p36.22	Migration and invasion inhibitory protein	regulation of mitotic progression	cancer (tumor suppressor)
*ADRB2*	5q32	Adrenoceptor beta 2	G protein-coupled receptor	nocturnal asthma, obesity, type 2 diabetes, cardiovascular disease
*ABLIM3*	5q32	Actin binding LIM protein family member 3	interaction with actin filaments	pain sensitivity
*NOD1*	7p14.3	Nucleotide binding oligomerization domain containing 1	role in innate immunity	asthma, inflammatory bowel disease, Behcet disease, sarcoidosis
*MSR1*	8p22	Macrophage scavenger receptor 1	macrophage-associated physiological and pathological processes	
*OR4L1*	14q11.2	Olfactory receptor family 4 subfamily L member 1	G-protein-coupled receptor	
*SSTR5-AS1*	16p13.3	SSTR5 antisense RNA 1		biased expression in cancer
*RNF43*	17q22	Ring finger protein 43	RING-type E3 ubiquitin ligase	mutations in colorectal and endometrial cancers
*C17orf58*	17q24.2	Chromosome 17 open reading frame 58	part of collagen-containing extracellular matrix	
*ZACN*	17q25.1	Zinc activated ion channel	zinc-activated ligand-gated ion channel	
*CYBC1*	17q25.3	Cytochrome b-245 chaperone 1	innate immune response	chronic granulomatous disease
*NARF-AS2*	17q25.3	NARF antisense RNA 2		
*SYNJ1*	21q22.11	Synaptojanin 1	phosphoinositide phosphatase	

Cited from the database “gene” in National Center for Biotechnology Information (NCBI) in U.S.A.

## Data Availability

The original data sheet, created and analyzed in the current study, is available from the corresponding author upon reasonable request.

## Supplementary Materials

The supporting information can be downloaded at https://www.mdpi.com/article/10.3390/ijms23158626/s1.
